# RETRACTION: Glycyrrhizin Attenuates Toll Like Receptor‐2, ‐4 and Experimental Vasospasm in a Rat Model

**DOI:** 10.1155/jimr/9817675

**Published:** 2026-03-03

**Authors:** Journal of Immunology Research

RETRACTION: C‐Z. Chang, S‐C. Wu, and A‐L. Kwan, “Glycyrrhizin Attenuates Toll Like Receptor‐2, ‐4 and Experimental Vasospasm in a Rat Model,” *Journal of Immunology Research* 2014 (2014): 740549. https://doi.org/10.1155/2014/740549.

The above article, published online on 23 July 2014 in Wiley Online Library (wileyonlinelibrary.com), has been retracted by agreement between the journal’s Editor‐in‐Chief Nadja Bakocevic; and John Wiley & Sons Ltd. A third party reported that images in Figure 1a in this article had been published in several other articles by some of the same authors. Each image was reported differently in the various publications. Additionally, the bands in Figures 6 and 7 were duplicated and rearranged, while both of these images display different experimental conditions.

The authors did not respond to an inquiry and request for original data by the publisher.

The retraction has been agreed to because the evidence of image duplication and manipulation fundamentally compromises the editors’ confidence in the results presented. The authors have been informed of the retraction.

